# Nanocuration workflows: Establishing best practices for identifying, inputting, and sharing data to inform decisions on nanomaterials

**DOI:** 10.3762/bjnano.6.189

**Published:** 2015-09-04

**Authors:** Christina M Powers, Karmann A Mills, Stephanie A Morris, Fred Klaessig, Sharon Gaheen, Nastassja Lewinski, Christine Ogilvie Hendren

**Affiliations:** 1 National Center for Environmental Assessment, Office of Research and Development, U.S. Environmental Protection Agency, 109 TW Alexander Drive, Research Triangle Park, NC 27711, USA; 2 Currently: Office of Transportation and Air Quality, Office of Air Quality, 2000 Traverwood Rd, Ann Arbor, MI 48105, USA; 3 RTI International, 3040 Cornwallis Rd., Research Triangle Park, NC 27709, USA; 4 Office of Cancer Nanotechnology Research, National Cancer Institute/NIH, 31 Center Drive, Bethesda, MD 20892, USA; 5 Pennsylvania Bio Nano Systems, LLC, 69 Homestead Drive, Doylestown, PA 18901, USA; 6 Leidos Biomedical Research Inc., Frederick National Laboratory for Cancer Research, 8560 Progress Drive, Frederick, MD 21702, USA; 7 Department of Chemical and Life Science Engineering, Virginia Commonwealth University, 601 W. Main St., P.O. Box 843028, Richmond, VA 23284, USA; 8 Center for the Environmental Implications of NanoTechnology (CEINT), Duke University, P.O. Box 90287, 121 Hudson Hall, Durham, NC 27708, USA

**Keywords:** curation, informatics, nanoinformatics, nanomaterials, workflows

## Abstract

There is a critical opportunity in the field of nanoscience to compare and integrate information across diverse fields of study through informatics (i.e., nanoinformatics). This paper is one in a series of articles on the data curation process in nanoinformatics (nanocuration). Other articles in this series discuss key aspects of nanocuration (temporal metadata, data completeness, database integration), while the focus of this article is on the nanocuration workflow, or the process of identifying, inputting, and reviewing nanomaterial data in a data repository. In particular, the article discusses: 1) the rationale and importance of a defined workflow in nanocuration, 2) the influence of organizational goals or purpose on the workflow, 3) established workflow practices in other fields, 4) current workflow practices in nanocuration, 5) key challenges for workflows in emerging fields like nanomaterials, 6) examples to make these challenges more tangible, and 7) recommendations to address the identified challenges. Throughout the article, there is an emphasis on illustrating key concepts and current practices in the field. Data on current practices in the field are from a group of stakeholders active in nanocuration. In general, the development of workflows for nanocuration is nascent, with few individuals formally trained in data curation or utilizing available nanocuration resources (e.g., ISA-TAB-Nano). Additional emphasis on the potential benefits of cultivating nanomaterial data via nanocuration processes (e.g., capability to analyze data from across research groups) and providing nanocuration resources (e.g., training) will likely prove crucial for the wider application of nanocuration workflows in the scientific community.

## Introduction

**Figure 1:**
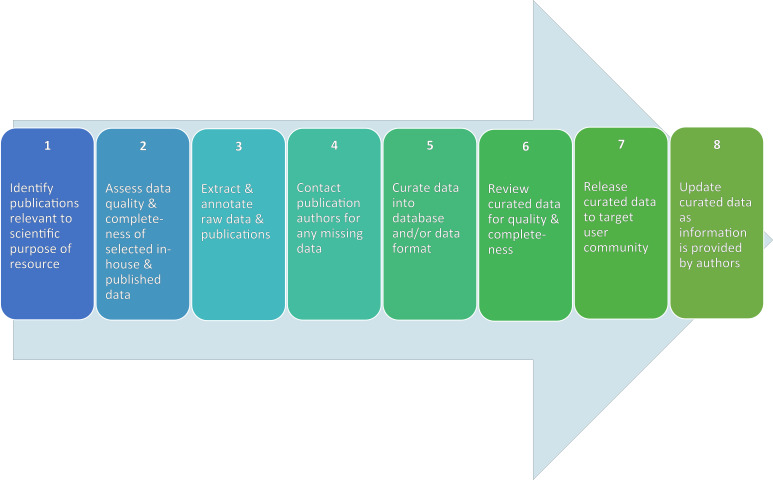
Common steps in nanocuration. The steps commonly included in nanocuration workflows are illustrated, including: 1) Identification of publications relative to the intended scientific purpose; 2) Preliminary assessment of data quality and completeness of selected in-house or publication data for data quality and completeness (with assumption that any in-house data would be pre-identified within a project prior to the wider publication search referred to in Step 1); 3) Data extraction of raw data and/or data from the publication; 4) Communication with publication authors; 5) Curation of data into the intended repository and/or data format (e.g., ISA-TAB-Nano) leveraging common data elements (CDEs) from relevant ontological resources (e.g., NanoParticle Ontology [NPO]); 6) Review of curated data for data quality and completeness; 7) Release of curated data; 8) Update of curated data as additional information is received from the authors. Though shown here in linear fashion, the order of these common steps for an individual process may be flexible and iteration is expected. The specific steps in a workflow may also differ across repositories depending on the intended purpose of the nanomaterial resource.

A tremendous growth in resources and tools to hold and organize large quantities of data has increased data availability to scientists, engineers, and others in the scientific community. Greater access to data repositories, data sharing platforms, and data visualization tools creates opportunities to compare and integrate information across a variety of diverse fields of study. For fields like nanoscience, or the study of materials at the nanoscale, this opportunity is particularly important given the wide array of disciplines that are inherently involved in synthesizing, testing, regulating, using, and developing new nanomaterial applications (e.g., chemistry, toxicology, ecology, risk assessment, material science). The complexity of developing tools for accessing, sharing, and viewing data relevant to nanomaterials has generated an entire field known as nanoinformatics. This paper is one in a series and focuses on a particular aspect of the nanoinformatics field, namely, the curation of data related to nanoscale materials (nanocuration) [1]. For this purpose, the experiences of three organizations (NCI, RTI and CEINT found in the listing of authors) were compiled into a questionnaire that was submitted to a further four organizations in order to describe current practices. Articles in this series are developed by the Nanomaterials Data Curation Initiative (NDCI), which is part of the National Cancer Informatics Program Nanotechnology Working Group [1]. Other articles in this series discuss several key aspects of nanocuration (temporal metadata, data completeness, database integration), while the specific focus of this article is on the nanocuration workflow, or the process of identifying, inputting, and reviewing nanomaterial data in a data repository (Figure 1).

## Discussion

### i. Importance and relevance of the workflow to nanocuration

A workflow is a critical component of nanocuration for several reasons. A workflow: 1) defines the process for data curation, 2) allows for comparison across data repositories to determine areas of standardization and bottlenecks, and 3) provides a consistent process for understanding the quality and completeness of a dataset [2]. Defining the process for data curation through the creation of a workflow presents an opportunity for individuals in an organization to establish and standardize the specific steps involved in identifying, inputting, and reviewing nanomaterial data for storage in the associated repository. A focused effort on each step in the workflow facilitates the identification of critical elements within and between each step, such as information transfers from one individual to another, quality control checks, and access rights necessary to input or review data. When individuals in an organization or institution document and define the data curation process, they not only create a valuable resource for future review, revision, and quality assurance/control (QA/QC) measures, but institutionalized workflows also facilitate the creation of training materials. Training materials in turn enable multiple curators to work in parallel, with a streamlined QA/QC process, and thereby mitigate redundant checking of curation decisions. This is critical to nanoinformatics progress, since curation (manual data entry or transfer from a data source) is the primary bottleneck to data collection once a repository structure and language are solidified. Related to the second aspect of the importance of a workflow, comparison between data resources, workflows serve as a written indicator of differences or similarities in underlying assumptions, order of operations, and standardization levels of, for example, data completeness. In comparing workflows from different data repositories, curators may identify common challenges (e.g., acquiring additional experimental design details from authors) or opportunities to leverage resources between repositories. In some instances, such workflow comparisons may lead to the use of common file formats, vocabulary, and structure. Common file, vocabulary, and structure conventions across data repositories in turn facilitates researchers and others utilizing data from across repositories in analyses. Finally, workflows facilitate researchers and other data users understanding the quality and completeness of the curated data. Indeed, in addition to the data quality support provided by the consistent curation practices of a defined workflow, the assessment of data quality and completeness is expressly included in two of the common curation steps articulated in Figure 1. Data quality and completeness is the topic of another article in this series and, thus, will not be discussed at length in this article. Nevertheless, understanding these concepts in various repositories is necessary for researchers or others using the data since different levels of quality or completeness are required for different uses of data (e.g., research prioritization, screening level decisions about hazard, quantitative risk assessment) [3–4].

### ii. Influence of organizational purpose or goals on design and application of a workflow

A discussion of a curation workflow requires an understanding of the curation purpose, (i.e., the objectives of the community sponsoring the data repository and the intended function of the repository). The diversity of communities and organizations involved with nanocuration reflects the multidisciplinary nature of nanotechnology. This diversity also has implications regarding workflow details for each separate curation effort, which inevitably involves validating data sources or characterizing the “quality” of data entries. The three examples that follow demonstrate the interplay.

For instance, the objective of the National Cancer Institute’s (NCI) cancer Nanotechnology Laboratory (caNanoLab; https://cananolab.nci.nih.gov/caNanoLab/) data portal is to provide a comprehensive resource for individuals in the biomedical nanotechnology research community to share data that supports the use of nanotechnology in biomedicine (e.g., novel cancer diagnostic or therapeutic tools and technologies). As part of NCI, caNanoLab uses a nanotechnology information object model (nano-OM) to capture standardized nanomaterial composition and characterization concepts [5]. The nano-OM facilitates the use of Common Data Elements (CDEs) for cancer nanotechnology research described in an established data format for nanomaterial data, NanoParticle Ontology [6] (The term Common Data Elements is used in particular by the National Institutes of Health (NIH) in describing their controlled vocabulary approaches, and refers to standardized data types that are consistent across datasets and resources). The use of the nano-OM in caNanoLab supports queries on publications, protocols, nanomaterials and associated compositions and characterizations. These data can be used by modeling and simulation tools to discover data patterns that guide decisions on new biomedical research directions and novel nanomaterials. Users can focus on particular nanomaterial(s) and biological phenomena through selection criteria for literature and research protocol sources that are curated into the repository. Based on the objectives of the repository, the workflow process must incorporate data and metadata (i.e., information about the data) related to: 1) nanomaterial physicochemical characteristics, 2) in vitro and in vivo assays that analyze nanomaterial properties, biological interactions, toxicity, or efficacy, and 3) information on the protocols used to analyze these nanomaterials and any associated publications.

In contrast, the purpose of RTI International’s Nanomaterial Registry (NR; https://www.nanomaterialregistry.org/) is to collect validated data from a broad field of accessible nanomaterial sources relevant to not only medical applications, but also the environmental implications of nanomaterials and their impact on human health and safety. While selection criteria regarding data sources remain a necessary element to the curation workflow, the NR uses an internally defined compliance score (minimal information about nanomaterials [MIAN]) to communicate the relative extent of physicochemical test data completeness to users [7]. This workflow process allows the NR to convey data quality information without restricting the incorporation of data into the repository due to a lack of information on experimental design, conduct, or outcome reported in the literature.

Finally, the Center for Environmental Implications of NanoTechnology (CEINT; http://www.ceint.duke.edu/) generates a wide array of nanomaterial data including characterization of pristine and naturally transformed particles, fate and transport data, toxicity data, and information on ecological impacts not limited to toxicity (e.g., nutrient cycling impacts) from laboratories within the Center and from collaborators. These laboratories represent a variety of scientific disciplines and use or develop well-founded, yet innovative procedures that may eventually be standardized. The CEINT-NIKC (CEINT NanoInformatics Knowledge Commons) focuses on developing the infrastructure and data gathering practices necessary to capture the full value of the Center’s multidisciplinary activities for integration and analysis not only of internally generated data, but also with any relevant literature that can also be curated into the system. The expectation is that some of the critical data may reside beyond publicly available peer-reviewed articles, and thus may need to be solicited directly from researchers (e.g., via theses, lab notebooks, spreadsheets). In this case, the primary selection criterion for including data in the repository is that the data are directly relevant to the driving research questions of the Center. The driving research questions focus on: 1) elucidating the characteristics of materials and systems, and 2) mechanisms driving nanomaterial behavior in complex systems; thus, data in the repository span a range of traditionally separate disciplines. Furthermore, the dynamic nature of nanomaterials in terms of changes in chemical identity as they migrate environmentally must be matched by an equally dynamic interaction of these disciplines in regularly evaluating both current and past data. This is not a matter of only data quality, but also of identifying new, useful concepts that bind the disciplines together for a common community purpose. The workflow process thus must be well-defined, yet flexible enough to incorporate new types of data or linkages across data types (e.g., dissolution rate at a particular pH and toxicity in a specific organism).

These three organizations (caNanoLab, NR, and CEINT-NIKC) differ in sourcing data to be curated (established protocols, literature sources, primarily internal or fully external), the intended users (medical researchers conversant with bioinformatics, the general nanotechnology public, and Center investigators), and function (modeling for repeatable experimentation, accessing nanomaterial sources, exploratory research requiring coordination among disciplines). For each, “high quality” means fit-for-purpose and thus the curation workflow is integral to meeting the community’s goal. The existence of established workflows in each organization allows for the identification of common challenges associated with the development or use of the workflow process. These challenges include: 1) establishing a minimal information set to include in the workflow, 2) determining a vocabulary (based on standards as much as possible) for the curators to use, and 3) defining how the data quality and validation are ensured in the workflow. In all three cases, the purposes of the repository necessitated that the workflow design include an opportunity to contact the investigators who developed the data (i.e., authors of peer-reviewed articles, Center members) in order to obtain complete and high quality data sets. In addition, the workflow can help facilitate sharing data across these or other resources. For instance, different organizations can incorporate a common data format in their respective workflows. An example data format is ISA-TAB-Nano, which is a file transfer protocol for querying among federated data repositories that are independently maintained by organizations with related, but not necessarily overlapping objectives [8]. Communication among federated repositories allows each separate community to tailor the workflow to their available resources, especially in this fluid period of debates regarding dose metrics, physicochemical characterization data sets, and protocol standardization.

Notably, in some organizations the term “curation” may be used in a less formal sense to simply describe the process used to identify data and integrate it into a data repository system. The process to formalize a curation workflow may take place after an initial phase of simply working through the informal process. The process of formalizing the curation workflow may be particularly important when a group expands or opens their repository to contributions from stakeholders outside of the research group. NanoDMS (http://biocenitc-deq.urv.cat/nanodms/), an FP7 project in the European Union, represents an example of using an informal curation workflow that may become more formalized during the group’s maturation. Ultimately, the purpose of the organization or group that develops the data repository not only drives the development of the workflow process, but may also determine how and when the workflow process is incorporated into the curation effort.

### iii. Established methods for workflows in mature fields

Organizations or groups that are working to incorporate or further develop a workflow for nanomaterial data curation may benefit from adapting methods established in other, perhaps more mature, fields (e.g., bioinformatics). In general, other fields utilize one of two approaches: 1) establish specific file formats with standardized vocabularies and fields, or 2) create collection formats at a generalized level to allow for the variation and uncertainty across a field. As a specific example of the first approach, the genomics community has developed a curation workflow that uses standardized file formats for both metadata and raw DNA sequence data for submissions into standard repositories [9]. A validation tool (Picard, http://broadinstitute.github.io/picard/) is then used to verify that the data fits the standard. An example of the second approach can be found within the *C. elegans* field with the WormBase repository (http://www.wormbase.org/#01-23-6). Notably, the genomics and WormBase workflows also take different approaches to the responsibility of entering data into a public repository. The genomics field requires authors to submit their own data using the provided file formats, whereas WormBase has a group of data curators responsible for identifying, entering, and managing data in the repository. Giving authors the responsibility of submitting data in standard formats to established repositories is an avenue for discussion in the nanomaterial community. Indeed, the NCI Alliance for Nanotechnology in Cancer now expects grantees to submit and share data using an established repository, caNanoLab (http://grants.nih.gov/grants/guide/rfa-files/RFA-CA-14-013.html). The extent to which other funding organizations add requirements for authors to share data in specified repositories will likely depend on a variety of factors, including the usability and accessibility of simple workflows for adding data to a repository.

### iv. Current practice in nanocuration workflows – Stakeholder responses to questions

**Figure 2:**
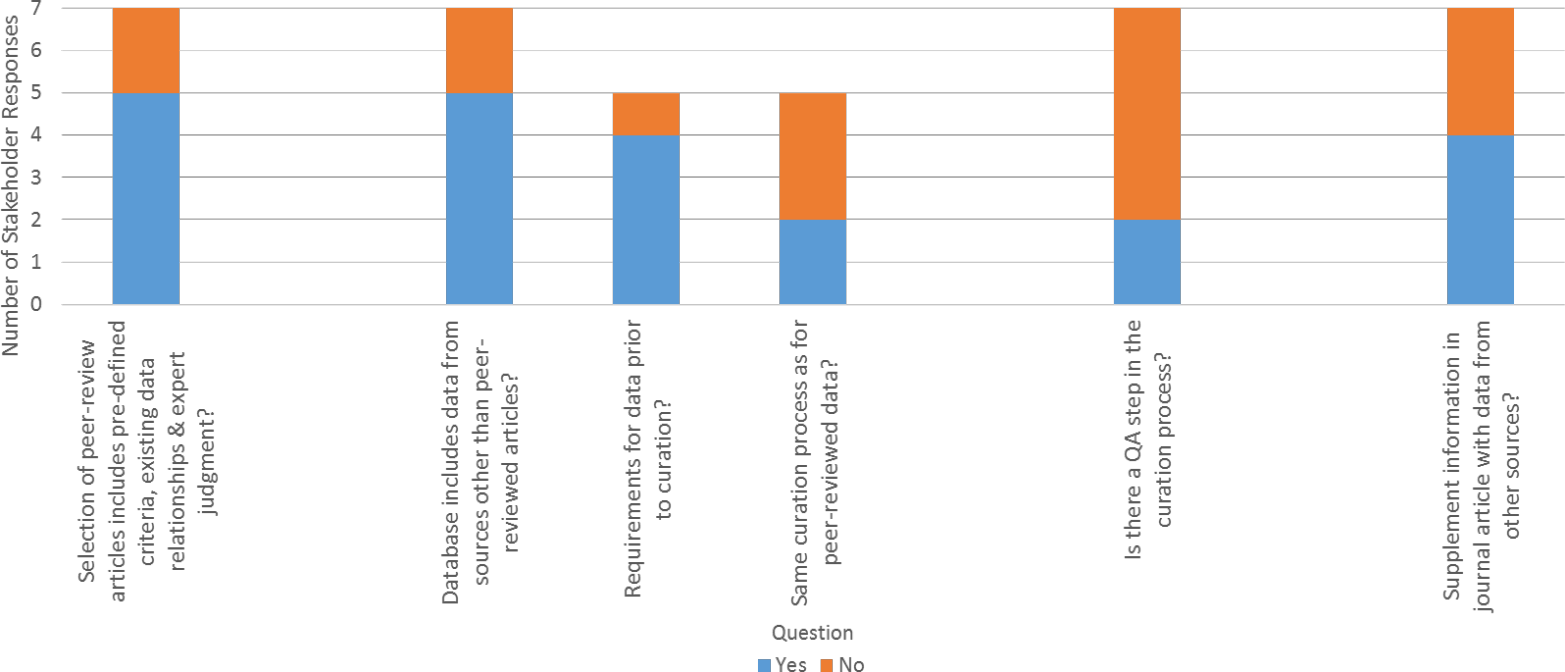
Stakeholder responses regarding sourcing Data. Stakeholder responses to questions related to sourcing nanomaterial data in a workflow for a data repository. Full text of stakeholder responses is available in Supporting Information File 1.

**Figure 3:**
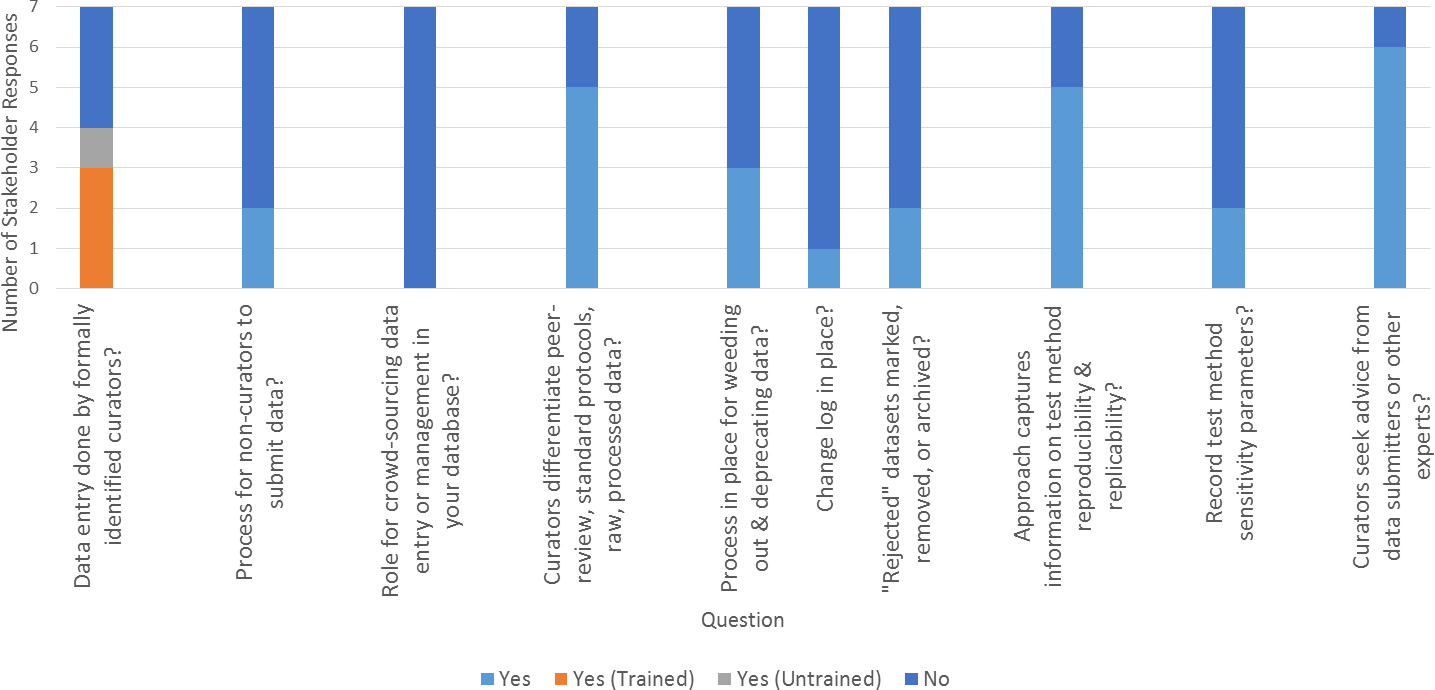
Stakeholder responses regarding data entry and review. Stakeholder responses to questions related to entering and reviewing data in a workflow. Full text of stakeholder responses is available in Supporting Information File 1.

**Figure 4:**
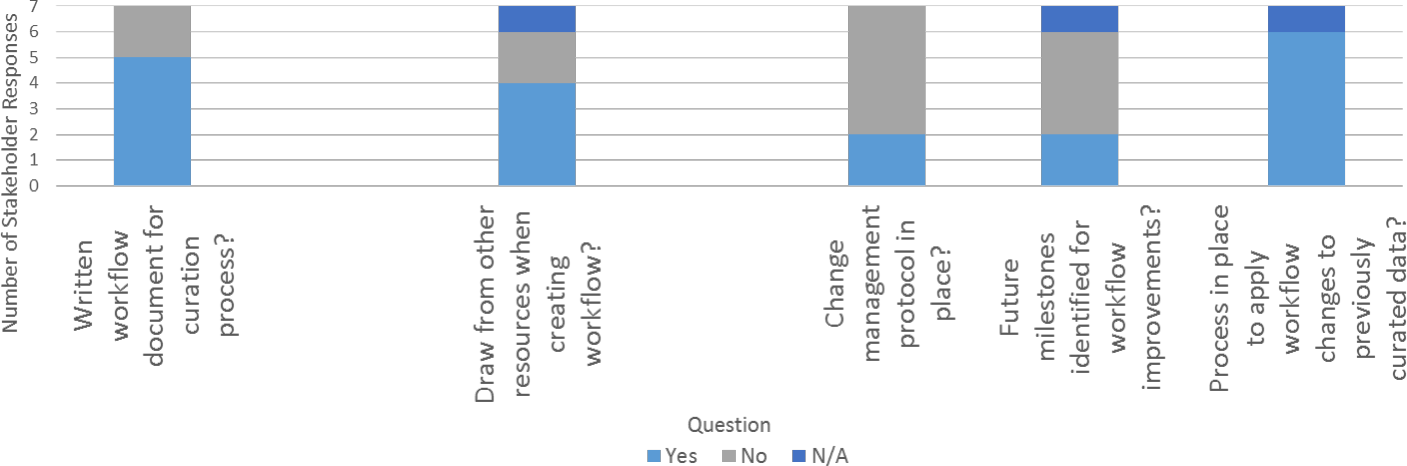
Stakeholder responses regarding creation and revision. Stakeholder responses to questions related to creating and revising a written workflow. Full text of stakeholder responses is available in the Supporting Information File 1.

**Figure 5:**
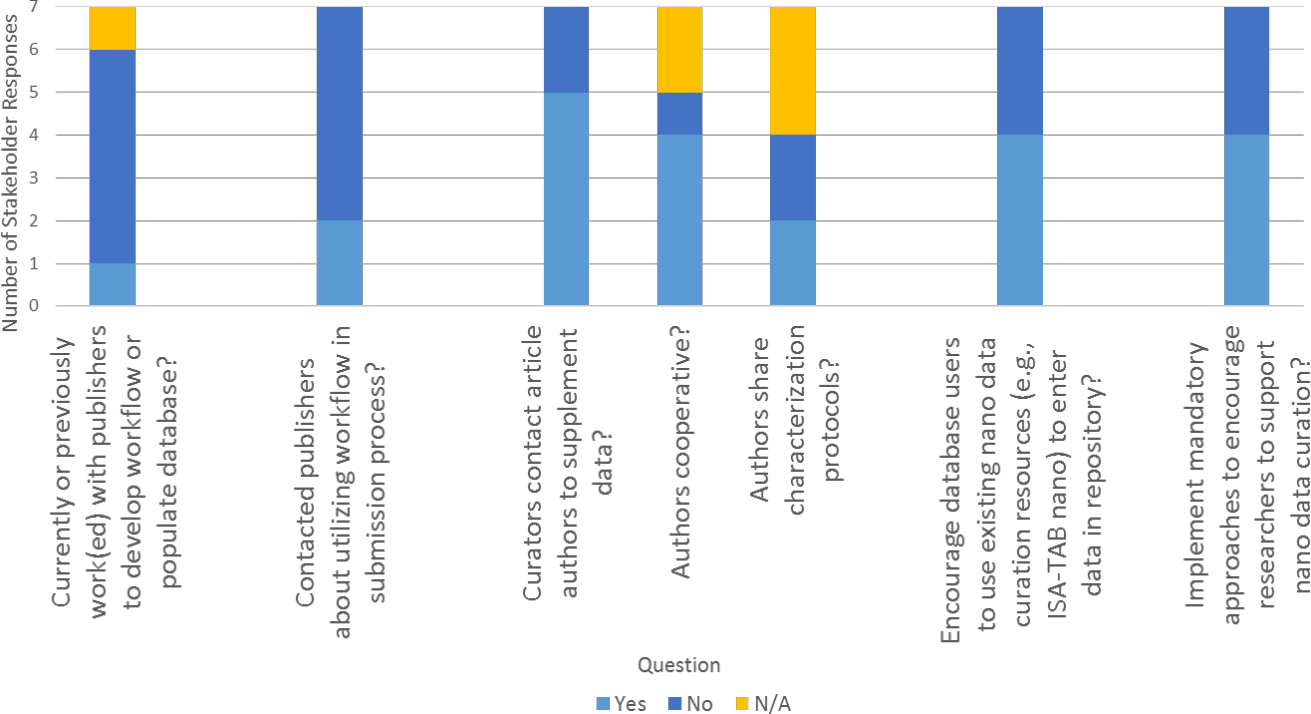
Stakeholder responses regarding working with other organizations. Stakeholder responses to questions related to interacting with other organizations to develop a workflow or populate a data repository. In each panel, the response categories (e.g., “yes”, “no”, “N/A”) for each question are provided in the legend. Questions are listed on the x-axis and the number of stakeholders responding in each category is on the y-axis. Full text of stakeholder responses is available in Supporting Information File 1.

To understand how practices in more established fields compare with the current state of nanocuration workflow practices across the field, the NDCI Leadership requested input from several individuals currently involved in developing nanomaterial data repositories. Seven representatives from organizations of different sizes and sectors (e.g., academia, government) responded to requests for input. Three of the respondents are also authors of this article since they represent organizations active in the nanocuration field. While the responding organizations represent a diverse swath of the nanomaterial field, the views presented here are not intended to provide a comprehensive representation of nanocuration workflows; rather, the intent of presenting these stakeholder responses is to help identify challenges and opportunities for improvement in nanocuration workflows by providing a snapshot in time of current practices. Additional details on the process used to contact and gain information from respondents is available in [1]. Briefly, the NDCI requested input from stakeholders in the fall of 2014 and winter of 2015 (November to January) on questions related to: 1) Sourcing data for nanocuration workflows, 2) Entering and reviewing data in a workflow, 3) Creating and revising a workflow, and 4) Interacting with other organizations to develop a workflow or populate their repository. Stakeholder responses are summarized below and in Figures 2–5 with additional details available in Supporting Information File 1.

#### a. Sourcing data for nanocuration workflows

As shown in Figure 2, two stakeholders consistently use established criteria for selecting data from the peer-reviewed literature to include in their repository, while four others report using loosely established, situation-dependent criteria. Most stakeholders (4 of 7) do supplement information in journal articles with information from other sources (e.g., searching for the paper in other databases) (Figure 2), since this approach provides a valuable source of supplemental data (see Supporting Information File 1 for details). When using sources other than peer-reviewed articles, stakeholders did consistently use established criteria (Figure 2). However, the majority of stakeholders (5 of 7) responded that their workflow does not currently include a quality assurance (QA) process. The two examples of a QA process included: 1) a manual review of data identified through a semi-automatic natural language processing (NLP) data extraction procedure, and 2) a second individual checking the initial curation (see Supporting Information File 1 for details).

#### b. Entering and reviewing data in a workflow

After determining how to source nanomaterial data for a repository, repository developers may establish guidelines for entering and reviewing data in the workflow. Of the stakeholders who responded to the NDCI request, just over half had individuals who are explicitly identified as a curator enter nanomaterial data (4 of 7 explicitly identified curators, with 3 of the 4 being specifically trained as a curator; Figure 3). In most cases, there was no process for non-curators to submit data to the repository (Figure 3). One example of a process for others to submit data consisted of researchers sending data in a standardized format (ISA-TAB-Nano) to a single person designated as responsible for data entry. Another stakeholder has a clearly defined and publicly available user’s guide for external submissions (see Supporting Information File 1 for details). Most respondents did note plans to develop a formal process for data submission in the future (see Supporting Information File 1 for details). All stakeholders distinguish peer-reviewed data from other types of information; however, not all further distinguish the data type (e.g., protocols, raw or unprocessed data) and some note that their repository only includes in-house data or only includes peer-reviewed data (Figure 3 and Supporting Information File 1). The majority of stakeholders (4 of 7) have a process in place to weed out or deprecate data, although they generally do not have a formal change log in place to document changes (only 1 of 7 stakeholders has a change log) and only two of seven explicitly mark and/or remove “rejected data” (Figure 3). Five of the stakeholders currently capture information related to test method reproducibility or replicability (Figure 3), though this typically occurs only through indirect measures (e.g., number of replicates, number of times protocol has been run in-house), or only in instances that data appear “interesting” (see Supporting Information File 1 for details). Only two of the stakeholders who responded currently capture information on test method sensitivity in completing their workflow (Figure 3); in one case this refers to the structural ability to incorporate sensitivity analyses if included in the publication, while in the other the functionality to carry out sensitivity analyses through query was part of the system design. In contrast, almost all stakeholders (6 of 7) consult advisors with relevant expertise if questions arise about data being entered through the workflow (Figure 3).

#### c. Creating and revising a workflow

As discussed in Section i (Importance of the workflow in data curation), there are a number of advantages to capturing the process for sourcing, entering, and reviewing data into a formal workflow. The majority of the stakeholders stated that they have written a workflow document to capture their process (5 of 7; Figure 4). These documented processes range in their formality and level of development; two stakeholders noted that they only recently developed a written workflow, while another stated that they were in the process of developing the documentation (see Supporting Information File 1 for details). The majority of stakeholders (4 of 7) reported drawing on other resources when creating their workflow. Most stakeholders (5 of 7) do not have a protocol in place to manage changes to their workflow (Figure 4), which might be expected since workflow documentation is in the early stages for this group of respondents. In addition, many (4 of 7) replied that they have not established specific future milestones for workflow improvements. In contrast, most stakeholders (6 of 7) did have a process in place to apply changes in the workflow to previously curated data (Figure 4). Such change processes seem particularly important in a field where the resource infrastructures and the curation processes are still in development.

#### d. Interacting with other organizations to develop a workflow or populate their repository

Efforts to work with publishers, journal article authors, and others involved in nanocuration can be beneficial in developing a workflow and populating a repository. However, based on stakeholder responses, it may be too early in the development of nanoinformatics infrastructures to see the establishment of such relationships. Respondents stated that there has been little activity to date in the nanocuration field to work with publishers on these issues, although there is recognition of the eventual importance of this aspect (Figure 5). One stakeholder did express interest in discussing the topic with publishers and noted that their organization includes individuals who serve as journal editors, which could facilitate such conversations (see Supporting Information File 1 for details). Compared to efforts to work with publishers, stakeholders indicated that there have been more efforts to contact journal article authors (5 of 7 stakeholders indicated they contacted authors; Figure 5). Yet, stakeholders who did make an effort to contact authors had dichotomized views of how willing authors were to share data or characterization protocols (Figure 5). Several stakeholders stated that authors were generally cooperative (but included caveats), while another stated that authors generally were not helpful. The respondent suggested that a lack of cooperation from authors could be due to a lack of interest in curating their data and/or the fact that authors were no longer in the same position (e.g., a PhD student generated data but had since graduated). One stakeholder noted that concerns about intellectual property rights might limit some authors’ willingness to share characterization protocols, while another suggested using established mechanisms to connect with researchers (e.g., the website ResearchGate) when requesting information from authors (see Supporting Information File 1 for details). In the longer term, curators could avoid the need to contact authors for additional information if researchers also reported their data using existing nanocuration resources (e.g., ISA-TAB-Nano) or other metadata tracking frameworks; however, only four of seven stakeholders stated that they encourage individuals to submit data in a standard format (e.g., ISA-TAB-Nano) (Figure 5). One reason that stakeholders provided for not using a standard format is that the data repository is only used in-house (see Supporting Information File 1 for details). To encourage more support for researchers to use nanocuration resources, stakeholders offered a variety of suggestions. Just over half of the stakeholders supported journals or funding agencies mandating that researchers use standard formats, while the other stakeholders emphasized the need for voluntary training or educational resources to encourage researchers to invest the time necessary for capturing their data in standard formats. Many stakeholders emphasized the need for significant funding to support the establishment and adoption of standardized data sharing mechanisms (Figure 5; see Supporting Information File 1 for details).

### v. Key challenges related to curation workflows for emerging and nanomaterials

**Figure 6:**
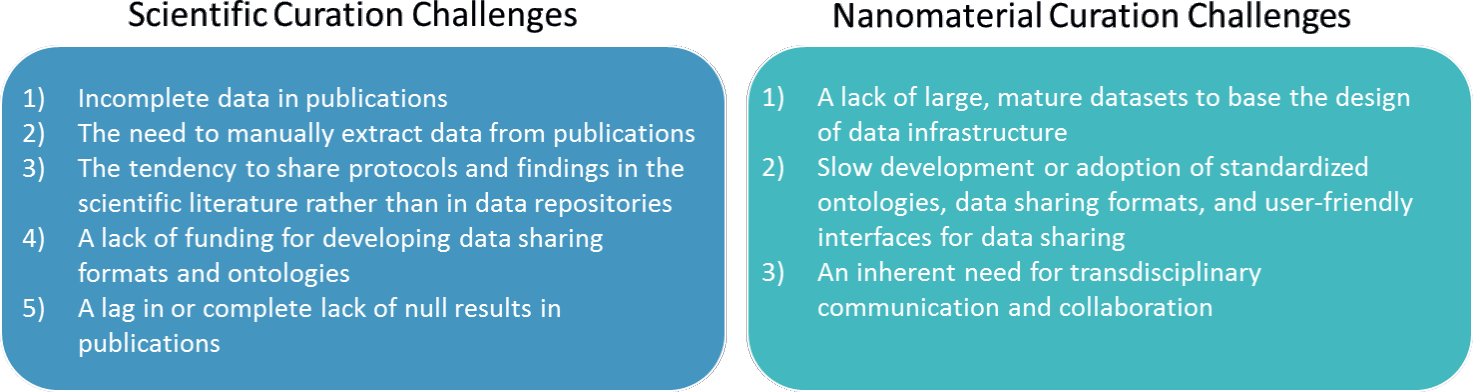
Scientific and nanomaterial curation challenges. Nanomaterial curation challenges expand on curation challenges inherent in general scientific curation.

While current practice in other, more mature fields provides some insight for the development of nanocuration workflows, the stakeholder responses described above indicate there are several challenges that the community will need to address in order to more efficiently and effectively develop nanocuration workflows. Some challenges are perhaps universally applicable to a variety of fields, both emerging and established, while others are more unique to emerging fields such as nanomaterials (Figure 6). Both types of challenges are discussed below in the context of what they imply for the development and application of data curation workflows in the nanomaterial community. The next section provides examples to illustrate the challenges outlined here.

Challenges that may impact a workflow and are generally applicable across the scientific community, include: 1) incomplete data in publications (i.e., an insufficient amount of information to reproduce an experiment or enable nanomaterial comparisons), 2) the need to extract data manually from publications, 3) a tendency to share protocols and findings in the scientific literature rather than in data repositories, 4) a lack of funding for developing data sharing formats and ontologies, and 5) a lag in or complete lack of null results in publications (i.e., journals rejecting manuscripts with null findings, or researchers not submitting data for publication until it includes at least one positive finding). These challenges generally impact how a workflow is or can be used (e.g., incomplete data in publications may require that the workflow include direct interaction with study authors to the extent possible). However, a workflow alone is unlikely to influence the scientific community to change its practices (e.g., investigators are unlikely to include additional data in publications because those data are required for one or more data repositories). To overcome these challenges in the nanomaterial community, and the scientific community more broadly, community members will need to understand the impact of current practices on data utility and applicability. Greater discussion between community members about the value of large data repositories and data sharing practices may have the greatest potential of driving toward resolution of these challenges. While the incentive of access to larger, interoperable datasets may encourage researchers and funding agencies to extend time, effort, and funds toward curating data into shared repositories, additional incentives will likely be necessary. As expanded on in Section vii, several incentives could drive researcher-contribution of data, including: 1) funds for data sharing by funding organizations, 2) requirements to submit data to central repositories from funding organizations or publishers, and 3) publication credit for dataset submission (e.g., receipt of a digital object identifier for data submissions). Ideally, these actions would be supported by data gathering software (e.g., electronic notebooks) that can export datasets in standard formats (e.g., ISA-Tab-Nano) and require minimal data restructuring by researchers. This would thus facilitate data curation that does not require a concerted effort separate from the research itself.

In contrast to broadly applicable challenges, challenges that are more unique to emerging fields, like nanomaterials, include: 1) a lack of large, mature datasets on which to base the design of data infrastructure, 2) slow development or adoption of standardized ontologies, data sharing formats, and user-friendly interfaces for data sharing, and 3) an inherent need for transdisciplinary communication and collaboration. Nanomaterial data workflows can likely facilitate progress in overcoming these challenges. For instance, by establishing and using a data curation workflow, caNanoLab, the Nanomaterial Registry, and CEINT-NIKC are all developing large data repositories that can guide the development of infrastructure for future nanomaterial data repositories as well as iterate improvements to themselves. The development and use of a workflow also inherently facilitates transdisciplinary communication and collaboration through the incorporation of data from a variety of domains (e.g., physicochemical, environmental transport, toxicity). Indeed, a workflow process is one aspect of a nanoinformatics approach that can actually be defined and followed in advance of a mature field, as a part of intentionally documenting research in pursuit of eventual data standardization. Seeing workflows as a critical part of overcoming some of the current challenges to nanocuration is perhaps one way to emphasize the importance of the nanomaterial community utilizing and further developing this integral piece of data curation. While nanocuration is being discussed in this section in terms of challenges, this effort is a response to the even greater challenge posed by the responsible development of an emerging technology that is fully expected to generate a large number of products and applications. Continuing the current tendency for each organization to maintain its own database with local interpretations of acceptable test protocols and data interpretation will impede the pace of innovation when organizations repeat work already done, but not accessible to others, or when firms and regulators are not aware of data pertinent to their discussions.

### vi. Examples of the identified challenges

Examples of the challenges outlined above help illustrate the importance of these issues and their impact on the goal of understanding nanomaterial interactions and behavior in different media. For instance, data curators at caNanoLab encounter several of the challenges outlined above, and these in turn impede the efficiency and effectiveness of the workflow. Related to the challenge of incomplete information in publications, caNanoLab curators have identified incomplete datasets, missing steps in protocol descriptions, and figures without underlying data or descriptions. Without these details, curators are unable to assess data quality and complete the curation workflow. In some cases, curators can obtain the missing information from study authors, but this slows the workflow process and is not always possible. Related to challenges more specific to the nanomaterial community, caNanoLab curators note that inconsistent terminology and a lack of automated data sharing tools impede the efficient implementation of their workflow.

Data curators at the Nanomaterial Registry have collaborated with CEINT-NIKC researchers to curate some of the Center’s findings into the Registry. While this collaboration will ultimately benefit the nanomaterial community by adding to the publicly-accessible repository, it actually highlighted some of the challenges outlined above. Specifically, CEINT-NIKC staff trained to curate the Center’s data into the Registry found that: 1) more data could be gathered when speaking directly to the researcher rather than relying on their publications (e.g., publications did not always share all of the physicochemical characterizations available on the nanomaterial tested, which were later captured by speaking with the researcher), and 2) in at least one case the original researcher had moved on from CEINT and targeted communication, with an associated time lag, was needed to retrieve additional information. Collaborators from both the Registry and CEINT concluded that curating from literature is not an optimal solution. This finding, and similar experiences across the nanocuration field, suggests that approaches like the NCI Alliance for Nanotechnology in Cancer that require authors to add data into a public repository may become more common practice moving forward.

### vii. Recommendations: Opportunities to leverage existing nanoinformatics resources for workflows and practical next steps for the nanomaterial community

Several opportunities exist to address the challenges discussed above in ways that leverage existing nanoinformatics resources. These opportunities can be broadly categorized in two areas: 1) to empower authors to submit data to repositories using standardized formats (e.g., ISA-TAB-Nano [8]) and nomenclature, and 2) to expand and further develop existing tools and repositories for nanomaterial data. Specific actions that the nanomaterial community can take to make progress in each opportunity area are outlined below to facilitate collaborative efforts in nanocuration.

Related to the first opportunity area, current practices in the nanomaterial community generally demand that curators of data repositories manually enter data from publications in the scientific literature. This practice not only slows down the workflow process, but also can frequently result in incomplete data entries or errors. To address this issue, the community could work to shift the responsibility of data sharing to investigators. Such a shift in responsibility could be spurred on by journal publishers and funding organizations requiring investigators to add their data to specified public repositories. In some instances, data could be added to repositories prior to publication during the data collection process in a non-public format, which could easily be made public later in an article. Entering data into repositories prior to publication could help reduce errors (i.e., minimize forgotten protocol details) and expedite the time to publication by avoiding the need to enter all the data at once, after completion of the study. If the repositories available for nanomaterial data develop methods to facilitate interoperability, then investigators could share their data with multiple stakeholder groups by entering information in a standardized format and ontology in one repository. This idealized scenario will of course take time to realize, but will only become possible through collaborative work in the nanomaterial community to support nanoinformatics. Some of that collaborative work might include the steps discussed below related to the second opportunity area: expanding tools and repositories.

Individuals and organizations in the nanomaterial community could consider mechanisms to enhance resources for development work on the ISA-TAB-Nano data-sharing tool and associated tools (e.g., time, opportunities for user community discussions, budgetary support). Development projects could focus on improving usability of the tool, automating some of the functions, and building data-entry interfaces. Resources for this work will be critical to support continued use of the tools, but to expand use of ISA-TAB-Nano and related tools, the community would benefit from opportunities for training. For example, a series of facilitated web-conferences (e.g., WebEx) or in-person workshops could provide valuable insight to new users. Resources for similar events that focus on more established users could support dialogue between data curators and ISA-TAB-Nano designers so that the tool continues to evolve in ways most useful to the user community. These discussions could also identify opportunities for workflow standardization across data repositories, as well as identify additional topic areas that would benefit from open dialogues in the nanocuration community. For instance, community users might discuss how natural language processing or other automated approaches might facilitate bringing data into repositories through ISA-TAB-Nano [10].

Recommendations proposed here have been based on the current landscape of the nanoinformatics field, and are focused on potential best practices to catalyze progress given the existence of multiple repositories and resources emerging from a variety of independently funded efforts representing diverse missions. It is not expected that a single unified resource for nanomaterial data analysis would ever be practical or particularly useful, given the established need for different levels of detail, data domains, and functionalities based on the driving purpose of the resource [1]. However, it may well be that some streamlining and optimization would be beneficial as the field matures, such that resources that have developed independently but that share similar analytical purposes, target communities, or sufficient CDEs might be merged into common resources to maximize effectiveness and sustainability.

## Conclusion

The curation workflow provides a means not only to share data through nanoinformatics, but also to communicate underlying assumptions about the data within and between organizations. The development and implementation of an explicit workflow process for nanocuration not only plays a role in building a single data repository, but also in providing information about standardization, common bottlenecks, and leverage points that can benefit the community as a whole. Current repositories and tools for sharing data provide a strong foundation for implementation of existing workflows such as those discussed above; however, progress in expanding the development and use of nanocuration workflows would benefit from efforts across the scientific community to address the myriad of challenges that face the implementation of nanocuration workflows (e.g., incomplete data in publications, funding for data sharing tools, use of standardized ontology). We welcome input from the nanomaterial community on the potential next steps to overcome the challenges laid out in this article, and encourage continued input as the effort moves forward. Interested community members can share feedback or join the National Cancer Informatics Program (NCIP) Nanotechnology Working Group by visiting https://nciphub.org/groups/nanowg/overview, and can learn more about the Nanomaterial Data Curation Initiative, in particular, by visiting https://nciphub.org/groups/nanotechnologydatacurationinterestgroup/wiki/MainPage.

## Supporting Information

Supporting Information contains all stakeholder responses that are summarized in Section iv (Current practice for nanocuration workflows: Stakeholder responses to questions) and Figures 2–5.

File 1 Stakeholder responses to Nanomaterials Data Curation Initiative (NDCI) questions regarding current nanocuration workflow practices (Note that respondents 5–7 are also authors on this article).
