# Supplementary material for: Nanocuration workflows: Establishing best practices for identifying, inputting, and sharing data to inform decisions on nanomaterials
Source: Beilstein J Nanotechnol. 2015 Sep 4;6:1860–71. doi: 10.3762/bjnano.6.189 (PMC4578434; doi:10.3762/bjnano.6.189)
Supplement: File 1 — Stakeholder responses to Nanomaterials Data Curation Initiative (NDCI) questions regarding current nanocuration workflow practices (Note that respondents 5–7 are also authors on this article). [file Beilstein_J_Nanotechnol-06-1860-s001.pdf]

Supporting Information
for
Nanocuration workflows: Establishing best practices for identifying, inputting, and sharing data to inform decisions on nanomaterials

Christina M. Powers[1,2], Karmann A. Mills[3], Stephanie A. Morris[4], Fred Klaessig[5], Sharon Gaheen[6], Nastassja Lewinski[7] and Christine Ogilvie Hendren[8*]

Address: [1]National Center for Environmental Assessment, Office of Research and Development, U.S. Environmental Protection Agency, 109 TW Alexander Drive, Research Triangle Park, NC 27711, USA, [2]Currently: Office of Transportation and Air Quality, Office of Air, Quality, 2000 Traverwood Rd, Ann Arbor, MI 48105, USA, [3]RTI International, 3040 Cornwallis Rd., Research Triangle Park, NC 27709, USA, [4]Office of Cancer Nanotechnology Research, National Cancer Institute/NIH, 31 Center Drive, Bethesda, MD 20892, USA, [5]Pennsylvania Bio Nano Systems, LLC, 69 Homestead Drive, Doylestown, PA 18901, USA, [6]Leidos Biomedical Research Inc., Frederick National Laboratory for Cancer Research, 8560 Progress Drive, Frederick, MD 21702, USA, [7]Department of Chemical and Life Science Engineering, Virginia Commonwealth University, 601 W. Main St., P.O. Box 843028, Richmond, VA 23284, USA and [8]Center for the Environmental Implications of NanoTechnology (CEINT), Duke University, P.O. Box 90287, 121 Hudson Hall, Durham, NC 27708, USA

Email: Christine Ogilvie Hendren* - christine.hendren@duke.edu

* Corresponding author

# Stakeholder responses to Nanomaterials Data Curation Initiative (NDCI) questions regarding current nanocuration workflow practices (Note that Respondents 5–7 are also authors on this article).

Workflow: Sourcing Nanomaterial Data

1. *For data curated from the peer-reviewed literature, how are publications selected?*
    - **Respondent 1:**
      Ad hoc basis; mainly down to individual reviewers. Final composites are edited by senior staff

    - **Respondent 2:**
      Currently, we select publications based upon a few different inclusion criteria. First, publications must be relevant to the nanoparticle of interest in our studies, poly(amido amine) (PAMAM) dendrimers. The next important criteria for our selection of publications is that the publication needs to focus on the use of PAMAM dendrimers to treat cancer.

    - **Respondent 3:**
      In general, two approaches are being followed:
        - A – Perform a web of science datasearch, based on a number of pre-defined keywords. Typically, a selection is subsequently made of the primary search results of the peer-reviewed literature to keep the further evaluation of the papers manageable, i.e. mainly to reduce the high number of papers typically retrieved.
        - B – A more specific search in a restricted number of Scientific Journals and grey literature. Searching of the grey literature is often done by means of a Google-search.

    - **Respondent 4:**
      NanoMILE are using primarily data generated within the project, but in cases where data from literature is used, depending on the type of data, and what it will be used for, more or less formal criteria are applied. For example, for developing QSAR approaches, publications were selected on the basis of providing sufficient variants of particles and unique datapoints for the modeling, rather than on the basis of being the most appropriately designed study for the purpose of in vitro toxicity assessment. In part, this was done by a single partner independently, and as a result of this, some tighter guidelines for selection of data to be utilized / curated within the project have now been developed (see below).

      We have not yet implemented a centralized database of publications that partners within NanoMILE are utilising and extrapolating information from, but that is something that we might consider, although given the diversity of end-points and

experimental approaches utilized within NanoMILE it is not clear what criteria could be applied that would represent excellence in study design across all. E.g. “omics” excellence might have very different requirements than particle ageing excellence, for example. Thus, we need to think a little more on this.

In the case of toxicity data such as LC_{50} etc., we apply the criteria developed within the ModNanoTox FP7 project, which assesses the data on the basis of the amount and type of NM characterization that has been performed and provides a total score on this aspect. Papers that pass this minimum threshold are then assessed for study design which includes things like use of controls, number of replicates etc. The detailed process is currently being written-up as a manuscript for publication.

- **Respondent 5:**
  Publications are selected based on established relationships with particular data resources. So, peer-reviewed literature, thus far has focused on, e.g., the works from a particular research center and studies performed on a particular manufacturer’s products or standard reference materials.

- **Respondent 6:**
  The criteria for determining whether a publication should be curated in caNanoLab includes: whether the publication is meaningful to the cancer nanotechnology field (cutting edge science), whether associated meaningful data is available in the publication or from the investigator, and whether the data is complete (e.g. contains material composition details and linkage information).

- **Respondent 7:**
  We select them based on whether they probe the research questions guiding our project, namely testing the predictivity of specific nanomaterial or environmental behavior parameters for outcomes of interest in complex environmental systems. If they test the same parameters that we are investigating in CEINT we consider the paper for curation.

2. *Are data from sources other than peer-reviewed literature (e.g., laboratories in your organization, online (public) databases, patents) entered into your database?*
   - **Respondent 1:**
     No

   - **Respondent 2:**
     No, only data from peer-reviewed literature is entered into our database.

   - **Respondent 3:**
     Yes, whenever suited data seem to be available, they are entered in the database. In such cases, the origin of the data is indicated.
   - **Respondent 4:**
     yes

- **Respondent 5:**
  Yes

- **Respondent 6:**
  Yes. Data from the NCI Nanotechnology Characterization Laboratory (NCL) is entered in the caNanoLab database. Also, additional data obtained from publication authors that is not included in the publication is obtained, when available. b.

- **Respondent 7:**
  Yes

2a. If so, do these data need to meet certain requirements prior to curation?

- **Respondent 3:**
  Yes – these are the same as for data retrieved from peer-reviewed publications

- **Respondent 4:**
  The main criteria is that data must be related to NanoMILE particles and include the unique NanoMILE particle and batch identifier, such that the data generated by partners can be linked to the appropriate characterization data, since in most cases the detailed characterization is being conducted by experts in nanomaterials characterisation, while the various toxicity, ecotoxicity, omics, exposure and ageing studies are being carried out by the relevant groups. Given this diversity, each Workpackage (WP) has been tasked with preparing a 1-page summary of minimum standards for data acceptability in their arena – i.e. number of replicates, appropriate dose-range determination, exposure conditions, controls, acceptable data variability, acceptable viability in controls etc. as a check-list for partners in planning their studies and assessing their data quality prior to including in the database.

- **Respondent 5:**
  Yes

- **Respondent 6:**
  Yes

- **Respondent 7:**
  Not uniformly; at this point we want to include all relevant data and use the datasets we receive to help us build the system that will best leverage the information.

2b.What are these requirements?

- **Respondent 3:**
  Essential is a proper description of the experimental conditions, including proper characterization of the physchem properties of the particles, chemical composition, etc. Thereupon, proper description of the test system used, and

description of the test guidelines according to which the test was performed. It also is important for high-quality data that possible deviations are well described.

- **Respondent 4:**
  As (a) above

- **Respondent 5:**
  The bare minimum for a data record in the Nanomaterial Registry is a description of the chemical composition and/or structure. But data sources have been prioritized which offer more data, such as particle size, and include measurement technique and protocol information.

- **Respondent 6:**

  Data entered must be from a reliable source and must be complete,

- **Respondent 7:**
  We do require that characterization of NMs and their surrounding media be included.

2c. *Does the curation process differ from peer-reviewed literature, and if so, how?*

- **Respondent 3:**
  No

- **Respondent 4:**
  Yes as here we specify a priori the quality aspects required, rather than assessing whether published data meets minimum standards for inclusion.

- **Respondent 5:**
  No, because the Registry curation process is conducted using a framework of fields and vocabulary which act as the guide for a curator when approaching any data source.

- **Respondent 6:**
  No. Similar to peer-reviewed literature, if the curator has additional questions, the data source will be contacted to clarify or provide additional details.

- **Respondent 7:**
  Yes. We may receive spreadsheets or emails with the data, if they are not from published sources. We go through these on a case-by-case basis to extract the pertinent information. This is not a high throughput process but a very tailored hunt for specific research-question-driven information.

3. *What is the Quality Assurance process to avoid transcription errors (e.g. converting units, etc.)?*
    - **Respondent 1:**
    Currently none

    - **Respondent 2:**
    Our process involves a semi-automatic natural language processing (NLP) data extraction method. We use our NLP method developed in house to associate numeric values with properties found in the literature. These values are then manually reviewed to insure the correct data has been taken from the literature. In the case of figures or tables, we have to use manual extraction to retrieve this data.

    - **Respondent 3:**
    Initially, no transcription is performed and the data are entered as reported. This includes the description of the units in which the data are reported. At a later stage we have the option to convert units.

    - **Respondent 4:**
    At present there is none specifically for the NanoMILE project, but discussions are underway with our knowledge hub partner, whose original expertise is in handling "omics" datasets but are now also handling nanomaterials-specific and toxicity datasets for the NanoMILE project, on what we can borrow or adapt from their toolbox to address this issue.

    - **Respondent 5:**
    The Registry uses a web-based curation platform for curation. Once a curator has transcribed the data from the data source, the record is promoted to another scientist for a quality assurance check. This fresh set of eyes, with thorough knowledge of the field and vocabulary requirements, will compare the Registry data record to the original data source. This person will make sure all possibly-curated data were captured and that it was transcribed accurately.
    - **Respondent 6:**
    The caNanoLab curator curates data into caNanoLab and re-reviews prior to making public. Due to resource constraints, there is no additional QA resource to review curated data. Data is linked to the originating source (publication, report - NCL) if an end user needs additional information

    - **Respondent 7:**
    The only QA in place now is that our lead curator, one of our primary post-docs developing and populating our CEINT-NIKC system, double checks his work. We have plans to develop some QA/QC tools we can use to identify anomalies in our data. When an anomalous value is identified, we plan to further examine it to determine if there was a reporting error, an entry error, or if a reported value is actually correct.

4. *Do you ever search in other databases to supplement the information in a journal article (for example, by searching that paper in other databases)?*
    - **Respondent 1:**
      No

    - **Respondent 2:**
      No we have never done this.

    - **Respondent 3:**
      Yes, and this is a valuable piece of information as it supplements the results of the other means of datasearching.

    - **Respondent 4:**
      Our knowledge base allows enriching of datasets and is linked to numerous other databases such as the nanoparticle ontology, omics platforms etc., but I don't believe that we have utilized it within the context of NanoMILE as yet to enrich / supplement data extracted from journal publications. However, this is something that we are certainly willing to try.

    - **Respondent 5:**
      Previously, the Registry has curated additional data from an article's supplemental material and has also received additional experimental details from the original researchers.

    - **Respondent 6:**
      The caNanoLab curator searches any supplemental information in which the publication author provides. This is typically images or additional articles. The curator does not search other databases unless they are referenced in the publication.

    - **Respondent 7:**
      Yes – we search the Nanomaterial Registry to see if they have additional information on the PCC for the material, to avoid double work. However, it should be noted that though the procedure employed for this process was used successfully to transfer and reformat the Nanomaterial Registry information into the database structure, it did not bring these data into full compliance with the CEINT-NIKC population protocols

Workflow: Entering and reviewing data

1. *Is any/all of the data entry for your resource carried out by formally identified curators? Are they expressly trained for this process?*
    - **Respondent 1:**
      Not currently

    - **Respondent 2:**
      Since we are the only ones using the data present in our "database," we have not formally identified a curator/s for the resource. I, David E. Jones, am the curator for

the resource currently. Since we developed our pipeline in house, I am trained as necessary for this process.

- **Respondent 3:**
  Yes, but only to a certain extent. Part of the work is carried out by a PhD student who by now is trained in data entry of 'nano-research'.

- **Respondent 4:**
  No. At the present time, data curation is by the researchers generating the data. However, some training for these persons, as part of their overall training would certainly be valuable and is something to consider.

- **Respondent 5:**
  Yes. However, in addition, the Registry has worked with research center (e.g., CEINT) representatives, training their scientists to enter their own data as curators.

- **Respondent 6:**
  The caNanoLab curator was formally identified by a university that has expertise in nanotechnology in biomedicine. The curator has skills supporting the curation of nanotechnology information in biomedicine and was trained in caNanoLab and ISA-TAB-Nano. The curator also participates in industry biocuration events.

- **Respondent 7:**
  Yes, and yes. The primary curator is the developer of the process. He directly trains any other curators, such as interns, in the process and double checks their curation for several publications before they curate on their own.

2. *Is there a process researchers and/or database users who are not curators for your resource to submit data to your resource?*
   - **Respondent 1:**
     At present there is no formal arrangement; we are currently in the process of creating a database containing meta-analysis from the literature and from our own experimental studies for SAR purposes but this is relatively low tech

   - **Respondent 2:**
     We haven't really thought about this yet or had anyone offer to submit data to our resource.

   - **Respondent 3:**
     No – not yet: this will happen in future.

   - **Respondent 4:**
     Given the size of the project, with 30 partners, each partner generating data has nominated one team member to be responsible for inputting / curating their data. Initially, the earliest datasets are beinge input by me (Iseult Lynch from UoB) to assess the process and see how easy / hard it is, and develop some guidelines / SOP

for data curation entry, which will then be passed to the nominated persons from each partner organisation. As per point (2a) we will likely have a short training session on this as part of our next consortium meeting.

- **Respondent 5:**
  No. The Registry is working toward external data submission but, to date, only use internal curators for data entry.

- **Respondent 6:**
  Yes. There are instructions for researchers and/or database users who are not curators to submit data into caNanoLab. Instructions are provided in the caNanoLab User's Guide and a video demonstrating data submission. Data submitted by users is not made publically available until the curator reviews the submitted data and makes the data publically available.

- **Respondent 7:**
  No. This capability is a next-phase activity for CEINT-NIKC (2016). We are working on creating tailored forms that allow self-curation from researchers directly. This would be checked for QA by the identified curators.

2a. If so, how many are there?

- **Respondent 4:**
  Given the size of the project, with 30 partners, each partner generating data has nominated one team member to be responsible for inputting / curating their data. Initially, the earliest datasets are beinge input by me (Iseult Lynch from UoB) to assess the process and see how easy / hard it is, and develop some guidelines / SOP for data curation entry, which will then be passed to the nominated persons from each partner organisation. As per point (2a) we will likely have a short training session on this as part of our next consortium meeting.

- **Respondent 6:**
- Currently, there are no users submitting data into caNanoLab. In the past, the NCL did submit some data. In the future, the Cancer for Nanotechnology Alliance members will be submitting data into caNanoLab.

2b. What is the submission process (e.g., create a form that is then reviewed by the database curators before being added to the database)?

- **Respondent 3:**
  We still need to figure this out.

- **Respondent 4:**
  All NanoMILE NMs have a unique number and this is associated with the nanomaterial tab of the ISATab format. Each investigation, study and assay is then linked to the relevant particle number(s) and the datasets are named according to a standardized approach, and the excel files or other datasets are then uploaded to the Knowledge base.

ISATab Forms are checked by the Knowledge base manager and queried with partner and project coordinator as needed, before being formally released and visible to the wider consortium. A key point is that no tab should be left empty, but instead should be filled with "Not applicable" if something doesn't apply or "not measured yet" if that is appropriate. Related datasets are then linked and uploaded, including image and raw files (or examples of such, plus details of how to request access to additional ones, where volume of data is prohibitive to upload all).

Note: this is likely to evolve as we get into uploading serious amounts of data from different WPs and addressing different aspects of the project.

- **Respondent 6:**
  The researchers submit nanotechnology samples, protocols, and publications into caNanoLab via web based forms. The researcher indicates that they would like to make the data publicly accessible. The curator reviews the data, makes any necessary changes and/or corresponds with the user, and changes the data access level to be publicly accessible.

2c. How do the formal curators exercise quality control? (these issues will be explored further in a subsequent paper focused on curator roles).

- **Respondent 3:**
  This too will be figured out for additional curators.

- **Respondent 4:**
  This is not established as yet, as we are still in a relatively early phase with data curation across the project.

- **Respondent 6:**
  The curators review submitted data as part of the QC checks.

3. *Is there any role for crowd-sourcing with regard to entering or managing data (entry, quality commentary, etc.) in your resource?*

- **Respondent 1:**
  No

- **Respondent 2:**
  Since our method is an NLP based approach, there really isn't a need for crowd-sourcing. Our goal is to create a tool, which independent researchers can build their database on-the-fly.

- **Respondent 3:**
  Not yet. This is however a future option, the more as we are involved in a couple of EU-funded projects in which data need to be entered by various curators (i.e. various project partners).

- **Respondent 4:**
  The Biomax knowledge Hub is regularly enriched with information from other database. Crowd-sourcing has not been used to date, but is something that could be considered. A specific use-case for crowd-sourcing that we can imagine is curation of text-mining results. In the field of cancer research the NCI and Biomax have collaborated to text-mine gene – cancer associations and subsequently manually verified all extracted associations manually. Such a task could be easily distributed and scaled and therefore would lend itself to crowd-sourcing.

- **Respondent 5:**
  Public commentary is available on each data record and comments are displayed at the bottom of the record’s page.

- **Respondent 6:**
  Currently, caNanoLab does not provide any facilities for crowd-sourcing other than general application support where users can report issues.

- **Respondent 7:**
  No.

4. *Do curators differentiate peer review, standard protocols, raw-to-processed data categories?*
   - **Respondent 1:**
     Yes, but these are not dedicated/trained curators

   - **Respondent 2:**
     Currently we are using only peer reviewed journal articles.

   - **Respondent 3:**
     Yes, although it should be noted that we are aware of the fact that peer review is not similar to sufficient data quality.

   - **Respondent 4:**
     Yes. To date most of the data is generated using NanoMILE protocols, and the protocols will also be linked with the ISATab Nano files and the datasets. We do make a distinction between the raw and processed data, and ideally request that both are submitted to the knowledge base. Resulting publications are then linked to the datasets also.

     As indicated above, we have not yet begun to compile / curate the literature data that partners are using, but will begin that process in the new year.

   - **Respondent 5:**
     Not explicitly at this time. However, indications are given in the data fields. For example, hyperlinks are provided for data sources and could be peer reviewed articles. Also, if a standard protocol was used, it is curated as the answer to one of the Registry’s best practice questions, “Standard protocol used?”

- **Respondent 6:**
  Currently, caNanoLab provides categorization for publications but does not categorize protocols and raw-to-processed data. As such, the curator does not differentiate standard protocols and raw-to-processed data; however, it would be beneficial to support data categories and associated standards to facilitate cross nanomaterial comparison and integration with analysis tools.

- **Respondent 7:**
  Yes.

5. *How does your workflow deal with weeding out and deprecating data?*

5a. Is there a change log with dates when deprecating data?
5b. Are "rejected" datasets marked, removed or archived?

- **Respondent 1:**
  A general review process consisting of expert PIs and Fellows with main data entry from PhD students

- **Respondent 2:**
  N/A

- **Respondent 3:**
  Yes!
  They are marked and separated from the other data, but they are not removed as there might be valuable information in them that is potential use in future.

- **Respondent 4:**
  NanoMILE distinguish 3 types of data changes.
  1. Updates of public databases linked into the NanoMILE knowledge base (NKB). In this case the versioning policy of the linked database applies
  2. Updates of data within the NKB retrieved from public sources with regular updates. Versioning information is added to all data updates that are part of the regular process. Data entries that are removed from the public sources are marked as "deprecated" and are archived
  3. Manually added data either from public sources, literature or project internal resources. No specific requirements and policy have been developed in the project so far. For metadata and low-throughput data an automatic versioning, archiving and change log are in place. For high-throughput data (HTD) a simplified management without versioning is currently used but versioning metadata could be associated with the HTD as well as archiving if required. Currently our experience from other collaborative research projects is that HTD data is shared only once a certain level of quality and stability has been reached which made archiving/versioning a low priority so far. If corrections had to be introduced it was mostly important to alert other partners.

- **Respondent 5:**
  The Registry does not weed or deprecate data. After being entered by curators, checked by QA, and reviewed by a third person (quality control) for proper scientific interpretation by the curator, the record is scored for amount of information based on the Registry minimal information about nanomaterials (MIAN). This is meant to express to the user the extent to which a nanomaterial was characterized and reported, thus, letting the user determine whether a record is usable to them or not.

- **Respondent 6:**
  The caNanoLab curation workflow currently does not weed out deprecated data. Additional time is spent up front identifying publications for curation based on the completeness of the data and other criteria (see question #1). Additional data is received from the author after curation and the workflow does include updating the data with additional data received by the author. a) Data is not deprecated; however, there is a date for when data is submitted into caNanoLab b) There are no rejected data sets as rejected data sets are not submitted into caNanoLab.

- **Respondent 7:**
  Though there is not currently a formalized process for this, the self-curation tool planned for future development will incorporate processes for both QA/QC and data rejection documentation. In the current database structure, the methods to handle there is a field that can be used to indicate the status of a data source that could be utilized to indicate if data are weeded out, as well as a place where notes can be made regarding subsets of data from that source.

6. *Do you capture information on test method ruggedness (replicability) and robustness (reproducibility)?*
   - **Respondent 1:**
     Some data that show interesting trends are followed up experimentally

   - **Respondent 2:**
     Currently we do not.

   - **Respondent 3:**
     Not really, these aspects are indirectly taken into account, as especially in peer-reviewed literature it is difficult to value these issues.

   - **Respondent 4:**
     As the NanoMILE project per se is mechanistically based rather than moving towards standardization or validation, this is not a very significant aspect. However, we do capture data regarding number of replicates, number of distinct times the protocol has been run, and towards the end of the project will, for each protocol / assay try to capture the information regarding replicability and robustness. Going forward, one approach might be to make an inventory of the NanoMILE assays and log where partners can indicate each time they use the assay, the number of replicates they performed, and note any unusual outcomes or whether the assay performed as

intended. This might be a useful way forward also in the specific cases of assays that we intend to propose for standardization at the end of the project, in order to capture this data in real-time rather than after the fact.

- **Respondent 5:**
  In our curation process, the Registry asks a set of best practice questions of the data source.
    i. Were raw data provided?
    ii. Proper controls used?
    iii. Instrument within calibration?
    iv. How many replicates were performed?
    v. Was a standard protocol reported?

- **Respondent 6:**
  caNanoLab supports the submission of characterization design and methods (unstructured text), and characterization techniques and instruments (structured); however, information is often not available in publications.

- **Respondent 7:**
  Our system has the structure to capture for example all experimental replicates individually as well as their average, so internal replicability to a single experiment is captured. Whether multiple experiments took the same measurement and how they compared is a primary goal, so it informs the queries we perform. Our future plans include the development of a compendium of methods that will include capture and comparison of detailed, queryable information on test methods.

7. *Is test method sensitivity to method parameters recorded?*
    - **Respondent 1:**
      No

    - **Respondent 2:**
      Currently it is not.

    - **Respondent 3:**
      No

    - **Respondent 4:**
      Not at present.

    - **Respondent 5:**
      The Registry is capable of curating the value of uncertainty reported with each measurement.

    - **Respondent 6:**

caNanoLab does not formally gather these types of data; however, if the published study includes experimentation to test ruggedness, sensitivity, etc., we accommodate these type of data in caNanoLab.

- **Respondent 7:**
  Sensitivity analyses are a primary goal of our query development and a driving goal of our system – amassing sufficient data to support such analyses is the rate-limiting step at the moment. Test methods for nanomaterial behavior and characterization are still in development, often by our center's research; preemptively capturing detailed information is often all that can be done at this stage of the development of nanoscience, to support this type of query as sufficient corroborating data emerge. The above-mentioned compendium would also address this point.

8. *Do curators seek advice from data submitters or from outside advisors or specific disciplines (data "approvers") when deciding on a data quality issue (which protocol, term or conclusion is to be favored)? If so, please describe roles or processes in place to enable this.*
   - **Respondent 1:**
     Not presently

   - **Respondent 2:**
     Yes, we work in conjugation with Hamidreza Ghandehari, PhD, and his laboratory group. This group is focused on nanoparticle drug delivery systems.

   - **Respondent 3:**
     Yes, they seek advice on an ad hoc basis. This includes advise of colleagues with relevant experience, consultation of the authors (usually via email), and consultation of experts in the field.

   - **Respondent 4:**
     As the data is generated within the NanoMILE project, and all partners are expert in their field, plus all datasets are discussed both at the WP level and at the consortium level (including with the external international advisory board), there is limited need at present for additional external advisors. However, should significant quality issues arise, we will certainly seek outside advice, including from the Nanosafety cluster WG on databases, the EU-US CoR on databases, ontology and modeling, and/or the NCI nanoWG. Indeed, we are keen to ensure that all emerging best practice is incorporated into the NanoMILE knowledge Hub and as such keep an active engagement in ongoing activities.

   - **Respondent 5:**
     The Registry ensures these levels of robustness in several ways.
     a. After quality assurance (transcription check), a data record is passed to a subject matter expert, who will then evaluate the curator's scientific interpretation of the original data source. This is important because the Registry uses a very strict parsing structure for nanomaterial data and sometimes a curator can make an

erroneous assumption about the relationships between information from the data source.

Also, in the design of the Registry's curation process, a minimal information was established to guide the curators. In order to capture the appropriate information about any given measurement technique (e.g., dynamic light scattering or field flow fractionation), workflow designers consulted subject matter experts on the measurement protocol areas necessary in order to report a repeatable measurement. These protocol lists became the minimal information on measurement techniques used by the Registry's curators and can evolve over time, depending on discoveries in the field.

- **Respondent 6:**
  The caNanoLab curator primarily seeks advice from the NCI Alliance for Nanotechnology in Cancer representative regarding data questions. The NCI representative corresponds with the publication author to obtain additional information or resolve quality related questions when needed.

- **Respondent 7:**
  Yes. Our curators spend time interviewing and clarifying with the CEINT researchers directly, and with discipline-specific experts within CEINT or with the corresponding author by email in the case of outside literature.

<u>Workflow Development: Creating and revising the workflow</u>

1. *Do you have a written workflow document for your curation process?*
   - **Respondent 1:**
     No

   - **Respondent 2:**
     We do have a written workflow document for our curation process.

   - **Respondent 3:**
     Yes, we do now, but this has only recently been established.

   - **Respondent 4:**
     Documenting the specific workflow for NanoMILE is in progress at present. As Biomax have multiple curation processes for a variety of data types NanomILE have adapted existing requirements and standards from MIBBI and ISA Tab as far as possible. These are documented as electronic forms for interactive input and format templates with corresponding format check scripts for data upload.

     For literature curation, corresponding existing documents from other projects (e.g. on cancer or allergy) can be provided by Biomax

   - **Respondent 5:**
     Yes

- **Respondent 6:**
  Yes. We maintain a Standard Operating Procedure (SOP) for caNanoLab data curation as well as workflow diagrams.

- **Respondent 7:**
  We have developed documentation that describes how the database should be populated. We currently allow the individual curator to determine the best way of getting data from the format in which the data are provided into the format needed for inclusion in the CEINT NIKC.

2. *Did your organization draw from or refer to other resources when creating this workflow?*
   - **Respondent 1:**
     N/A

   - **Respondent 2:**
     We did look at similar processes when creating our workflow.

   - **Respondent 3:**
     No, not specifically

   - **Respondent 4:**
     Biomax, who are managing / developing the Knowledge base with us, have long experience in managing datasets for "omics" and thus a lot of the workflow has been taken directly from their existing e-infrastructure, with the nano-parts being added via the collaborations within NanoMILE (specifically with the UoB team initially).

   - **Respondent 5:**
     The Digital Curation Center

   - **Respondent 6:**
     Workflow diagrams and the SOP were created from prior experiences in curating nanotechnology information. Resources used in the development of the caNanoLab curation workflow were in house.

   - **Respondent 7:**
     Yes. We looked extensively at the Nanomaterial Registry and ISA-TAB-Nano when developing our database population protocols.

3. *How do you deal with introducing changes to your workflow process?*
   - **Respondent 1:**
     Discussions with involved/relevant users

   - **Respondent 2:**
     We have not introduced any changes to our workflow process yet, however currently we are working on some text classification methods to improve the precision of our

NLP method. Upon completion, this will be added to the workflow process. There are also future plans to improve even further upon the current workflow.

- **Respondent 3:**
  See 3a - 3c

- **Respondent 4:**
  Changes are introduced by consensus development with all partners involved in a specific workflow. The workflow documents are versioned and consequences of changes for existing data are assessed and if necessary are applied to previously curated data. Currently these change discussions are induced by feedback from data providers, curators and users rather than a pre-defined workflow improvement plan.

- **Respondent 5:**
  The Registry has a Curation Index document that tracks curation rule changes, but the overall workflow has remained the same.

- **Respondent 6:**
  Workflow changes do not occur often and are typically reviewed during team meetings with the NCI and Curator. For example, the curation of ISA-TAB-Nano files was added to the workflow and reviewed during the team meetings. a) There is no formal Change Control Board (CCB). The team is relatively small. b) There are no future milestones planned for workflow improvements. c) Prior workflow changes did not require a change in the current methods for data capture other than additions where needed. For example, providing a DOI based URL was added to support a bi-directional link to a publication vendor. This required an addition of a URL for some of the publications but not all. As such, the curator added the URLs to the impacted publications.

- **Respondent 7:**
  See 3a - 3c

3a. Do you have a change management protocol?

- **Respondent 1:**
  No

- **Respondent 3:**
  No.

- **Respondent 7:**
  Not currently, but, if we get to the point where we have a more establish method of collecting data from researchers, and establish a more formalized workflow, we will consider how to manage workflow changes.

3b. Do you have future milestones planned for workflow improvements?

- **Respondent 1:**
  Not presently

- **Respondent 3:**
  No - no specific milestones.

- **Respondent 5:**
  Yes, smart curation features should soon mitigate the need for a QA step in our workflow. At that point, QA will not need to check grammar or data entry as much and will just focus on whether or not the curator captured all of the available data from the source.

- **Respondent 7:**
  Yes.

3c.How are changes in workflow applied to previously curated data (e.g. a change from "check if the data uses units" to "check if the data uses normalized units")?

- **Respondent 1:**
  Reviewed as necessary when performing statistical analysis

- **Respondent 3:**
  This is done on an ad hoc basis: the whole database is re-evaluated in such a case and the exact procedure to be applied is selected based on the changes needed.

- **Respondent 5:**
  Thus far, changes in curation rules have been easily dealt with. Our database administrator has been able to update terminology by programing a mass correction into the data.

- **Respondent 7:**
  One of our primary goals in populating the CEINT NIKC is that we follow strict protocols that will allow us to query our database in meaningful ways. We are striving for consistency. If changes in workflow are needed, we hope to be able to make changes to existing data in the database in a systematic way so that consistency will be maintained

<u>Workflow Collaborations: Efforts to interactively work and connect beyond your organization within the scientific community</u>

1. *Do you currently or have you previously work(ed) with publishers to develop your workflow or populate your resource?*
   - **Respondent 1:**
     No

   - **Respondent 2:**
     We have not worked with publishers to develop our workflow or populate our resource.

- **Respondent 3:**
  No

- **Respondent 4:**
  Currently working with Scientific data to publish an initial version of the database and its description and purpose. This is going more slowly than planned though due to competing demands for time.

- **Respondent 5:**
  No

- **Respondent 6:**
  Yes. We are currently working with publishers to add a bi-directional link between caNanoLab and the publication vendor for nanotechnology publications that have been curated in caNanoLab. The bi-directional link is expected to be in place by Q1 2015. We are also working with individual journals to promote the availability of our repository for data deposition.

- **Respondent 7:**
  No. We have discussed this in informal conversations with multiple editors at this preliminary stage, and do see this as an eventual possibility.

2. *Have you been in contact with journal publishers regarding their interest in utilizing nanomaterial data curation workflows for their submission processes?*
   - **Respondent 1:**
     No

   - **Respondent 2:**
     We have not been in contact with journal publishers regarding their interest in utilizing nanomaterial data curation workflows for their submission processes.

   - **Respondent 3:**
     No, although there is involvement in general terms in curation aspects for specific journals: we provide assistance in this respect to specific journals based upon our knowledge gained so far.

   - **Respondent 4:**
     No, but would be happy to support any ongoing efforts to this end.
     NanoMILE has two journal editors in the consortium – Flemming Cassee (Particle & Fibre Toxicology) and Hakan Wallin (Nanotoxicology) so this is certainly something that we could consider / discuss / debate easily.

   - **Respondent 5:**
     No

- **Respondent 6:**
  Yes; however, the scope has primarily been getting the publication vendors to recommend caNanoLab as a resource for submitting data associated with the application of nanotechnology in biomedicine.

- **Respondent 7:**
  Yes. At a later stage of nanoinformatics development, with more data maturity and protocol standardization, this seems to be a potential route to leverage and integrate large quantities of data.

3. *Do curators contact authors of journal articles to supplement information regarding materials they used, characterization process, meta-data needs, or other aspects of the article?*
   - **Respondent 1:**
     Yes

   - **Respondent 2:**
     We have not done this.

   - **Respondent 3:**
     Yes, but only to a limited extent

   - **Respondent 4:**
     We have not tried this. Although if we were to, I imagine we would have more success if the scientists initiated contact, e.g. via ResearchGate or other sharing resource.

   - **Respondent 5:**
     Not always. This has only been done at the Registry when we already know the author of the work (i.e. they directed us to the article).

   - **Respondent 6:**
     The NCI Alliance for Nanotechnology in Cancer contacts authors on behalf of the curation. a) Authors have been cooperative – especially authors involved in the NCI Alliance, b) Authors share additional characterization details but do not typically share the detailed protocols

   - **Respondent 7:**
     Yes.

   3a. Are authors cooperative?
   - **Respondent 1:**
     On the whole, yes.

   - **Respondent 3:**

No – not really. The main problem is that not all authors have an interest in this topic, or (for instance in case of PhDs) have left.

- **Respondent 5:**
  Yes, but perhaps because of the established relationship.

- **Respondent 7:**
  An advantage of beginning with an integrated established Center is that we have ongoing relationships to draw upon. A major focus of CEINT-NIKC development has been to base the progress upon efforts that will help address research questions our member scientists are interested in, so that we create a value-added resource for the contributors rather than simply an additional task by asking them to participate and eventually self-curate.

3b. Do authors share characterization protocols?

- **Respondent 1:**
  Often, but authors can understandably be wary of sharing to much information which may impact on intellectual property. Ourselves and our collaborators ensure that such things are protected prior to dissemination.

- **Respondent 3:**
  Depends: sometimes they do, sometimes they don't.

- **Respondent 7:**
  Yes, but often we only get the detail we need via interviews with the curator.

4. *Do you encourage database users to utilize existing curation resources for nano (e.g., ISA-TAB nano)?*
   - **Respondent 1:**
     Not currently
   - **Respondent 2:**
     We do not, as our tool is only being used in house.
   - **Respondent 3:**
     No, not yet.
   - **Respondent 4:**
     Yes, NanoMILE has adopted ISA-TAB nano as the standard format for all data to be submitted to the NanoMILE knowledge hub.
   - **Respondent 5:**
     Yes
   - **Respondent 6:**

caNanoLab encourages users to submit data into caNanoLab and the NCI is requiring data sharing in the Investigator's NCI Alliance Data Sharing Plan. Currently, the caNanoLab curator creates ISA-TAB-Nano files and encourages the use of ISA-TAB-Nano; however, additional tools are needed to facilitate the auto-generation of ISA-TAB-Nano files from caNanoLab –or- to allow users to easily create ISA-TAB-Nano files via extended ISA-TAB tools which provide links to ontologies.

- **Respondent 7:**
  Yes, we automate this encouragement by incorporating aspects of these resources (e.g. established vocabulary for specific parameters) into CEINT-NIKC development wherever possible.

5. *How do you think researchers could be encouraged to support nano data curation (e.g., public grant awardees required to provide certain final reporting outputs, such as ISA-TAB nano files or Nanomaterial Registry entries)?*

- **Respondent 1:**
  Workshops or general online resources explaining the merits fo such standardization as well as how they may be implemented to curation "naïve" groups.

- **Respondent 2:**
  It would be a great step if researchers would be required to submit ISA-TAB nano files or other related documents, much like is currently being done in protein research, with the PDB. Another option would be to create a standard format in which nanotechnology journal articles are written. This would drastically improve the ability to use NLP methods to mine text. Both of these options would definitely propel the field of nanoinformatics, and nanotechnology in general.

- **Respondent 3:**
  I think that above all it is to be considered that supporting nano data curation is a voluntary action that will take time of any researcher. In practice, researchers are reluctant to spend time on something that they might consider not to be of primary importance to them. ISA-TAB nano is important in this respect, as are obligations to nano data curation posed upon authors by Journals. It is to be noted that both roles differ by definition: whereas the role of ISA-TAB nano is especially related to assisting in the assessment of the proper information to be provided and providing the backgrpoun to the need to do so, the Journals have the 'power' to impose curation actions and actually implement them. An important role of ISA-TAB nano is therefore to educate scientists and show the importance of data curation.

- **Respondent 4:**
  Journal and/or funding agencies requiring all datasets to be reported / deposited and the format for this would be the ideal, but of course this would require that there be agreed place(s) to deposit the datasets and full agreement regarding reporting format. However, since most groups seem to be converging on ISA-TAB nano the latter should not be an issue.

- **Respondent 5:**
  Encouragement/requirements are needed from journals and/or funding agencies.

- **Respondent 6:**
  A pilot study should be initiated in each of the nanotechnology domains (e.g. biomedicine) that involves the collection of data sets to support a specific scientific use case (e.g. effects of nanomaterial size and shape on biodistribution). The data can then be efficiently analyzed and results published demonstrating the value of data sharing to researchers to encourage data submission. In addition to this, public grant awardees should be required to submit data into the associated repository (caNanoLab for biomedicine). The submission tools should be designed to support the import and export of ISA-TAB-Nano files to give researchers the option of web based submission vs. ISA-TAB-Nano submission.

- **Respondent 7:**
  We believe they can be encouraged to support this only if they are provided with clear communication and tools that communicate the motivation for, and decrease the workload of, sharing/integrating data. Significant funding to research and develop such infrastructure and communication will be required, along with community-wide demand for such detailed reporting such as establishment of journal submission requirements.
